# Hierarchical Graph Representation of Pharmacophore Models

**DOI:** 10.3389/fmolb.2020.599059

**Published:** 2020-12-14

**Authors:** Garon Arthur, Wieder Oliver, Bareis Klaus, Seidel Thomas, Ibis Gökhan, Bryant Sharon, Theret Isabelle, Ducrot Pierre, Langer Thierry

**Affiliations:** ^1^Department of Pharmaceutical Chemistry, University of Vienna, Vienna, Austria; ^2^Inte:Ligand Software-Entwicklungs und Consulting GmbH, Vienna, Austria; ^3^Institut de Recherches Servier (IdRS), Croissy-sur-Seine, France

**Keywords:** pharmacophore modeling, protein structure, clustering, human glucokinase, hierarchical graph representation, protein ligand binding, molecular dynamic (MD) simulation, virtual screening

## Abstract

For the investigation of protein-ligand interaction patterns, the current accessibility of a wide variety of sampling methods allows quick access to large-scale data. The main example is the intensive use of molecular dynamics simulations applied to crystallographic structures which provide dynamic information on the binding interactions in protein-ligand complexes. Chemical feature interaction based pharmacophore models extracted from these simulations, were recently used with consensus scoring approaches to identify potentially active molecules. While this approach is rapid and can be fully automated for virtual screening, additional relevant information from such simulations is still opaque and so far the full potential has not been entirely exploited. To address these aspects, we developed the hierarchical graph representation of pharmacophore models (HGPM). This single graph representation enables an intuitive observation of numerous pharmacophore models from long MD trajectories and further emphasizes their relationship and feature hierarchy. The resulting interactive depiction provides an easy-to-apprehend tool for the selection of sets of pharmacophores as well as visual support for analysis of pharmacophore feature composition and virtual screening results. Furthermore, the representation can be adapted to include information involving interactions between the same protein and multiple different ligands. Herein, we describe the generation, visualization and use of HGPMs generated from MD simulations of two x-ray crystallographic derived structures of the human glucokinase protein in complex with allosteric activators. The results demonstrate that a large number of pharmacophores and their relationships can be visualized in an interactive, efficient manner, unique binding modes identified and a combination of models derived from long MD simulations can be strategically prioritized for VS campaigns.

## Introduction

Understanding the biomolecular recognition of ligands and their interactions with macromolecular targets is of utmost importance for the successful discovery of novel biologically active compounds (Fenwick et al., 2011). One way to approach this problem in drug design is the modeling of ligand-target interactions as pharmacophores. Pharmacophores are defined as an ensemble of steric and electronic features that is necessary to ensure the optimal supramolecular interactions with a specific biological target and to trigger (or block) its biological response (Wermuth et al., 1998).

In general, pharmacophore models are either derived from ligand-target complexes (structure-based) and/or a set of known active molecules (ligand-based) and can then be used as queries for an *in silico* virtual screening (VS) to find compounds with similar stereoelectronic features (Langer, 2010; Leach et al., 2010; Schuster, 2010). One limitation of structure-based (SB) modeling is that all possible interactions between a target-ligand complex may not be captured since they are derived from static representations. The fact that proteins are flexible structures and interactions with ligands are inherently dynamic is well-known and remains to be an important problem with emerging *in silico* solutions in various contexts (Cozzini et al., 2008; Boehr et al., 2009). Molecular dynamics (MD) simulations have recently been used to sample possible protein conformations (Durrant and McCammon, 2011; De Vivo et al., 2016; Liu et al., 2018) which were then used to derive multiple pharmacophore models from an initially static crystallographic structure. Choudhury et al. (2015) generated 3-D pharmacophore models from each snapshot of a MD simulation and selected the best performing model after docking and VS rescoring. The selection of a single “best performing” pharmacophore model was also pursued by means of clustering (Sohn et al., 2013; Spyrakis et al., 2015), providing better VS results than “classical” x-ray crystallographic derived structure-based pharmacophore models. However, to determine the “best performing” model requires datasets of known active and inactive compounds to assess the performance of the models. In cases with new targets during early hit finding stages, this information may be yet not be available and prioritizing pharmacophore models for VS campaigns can be challenging.

To overcome the need to select one unique representative set of pharmacophore models, Wieder et al. (2017) developed the “Common Hits Approach” (CHA) in which multiple 3D pharmacophore models derived from a MD simulation were partitioned according to their feature compositions and used for subsequent VS runs. A single final hit-list was obtained using a consensus scoring function to rank and combine the screening results which were originally obtained for each unique model enabling a prioritization of virtual hits based on a set of MD derived models. Recently, Polishchuk et al. (2019) improved the workflow by adapting the consensus scoring function to consider the number of conformations of each molecule retrieved by the VS runs. Based on these studies, Madzhidov et al. (2020) analyzed the performance of a set of pharmacophore models and developed a probabilistic approach for consensus scoring, leading to a method which is less sensitive to the poor performing models in the pool. Although these consensus-based approaches provided better results than a “classical” pharmacophore approach, they demanded considerable computational resources due to the required multiple VS runs.

Nowadays, MD simulations allow for a thorough sampling of the conformational space—even of large biological systems and the generation of structure-based pharmacophore models is no longer limited to single crystallographic structures. As a direct result of the improvement of modern hardware, most computation laboratories can now perform MD simulations at the nanosecond scale in a few hours. However, performing consecutive VS runs on very large libraries (millions of compounds) is still a crucial time limiting factor. To address this issue, this paper presents a hierarchical graph representation of pharmacophore models “HGPM,” which aims at the easy to comprehend visualization of pharmacophore model related information and thus can greatly aid in the prioritization and selection of pharmacophore models for subsequent processing steps. While previous works reduced the number of pharmacophore models by clustering crystallographic structures or 3D pharmacophore information, this graph representation focuses on the view of hierarchical pharmacophore feature information to support the users in the model selection process in order to reduce the number of models for ensuing VS runs. A single representation of multiple pharmacophore models, for example, derived from an MD simulation, has several advantages: (i) The introduction of an easy to comprehend graph-based view of all unique models and their relationship, that were observed (Maggiora and Bajorath, 2014; Métivier et al., 2018). (ii) A simpler, less error-prone selection process of 3D pharmacophore models especially for long MD simulations for virtual screening runs. (iii) The possibility to expand the displayed information by the addition of models generated from other systems or MD simulations.

The following sections will focus on the algorithmic details and the computational procedure for the generation of the hierarchical graph representation of pharmacophore models. Furthermore, the methodology will be demonstrated and discussed in the context of the human hexokinase IV as a case study, illustrating how pharmacophore information derived from MD simulations can be displayed and put to good use with this approach.

## Materials and Methods

### Protein–Ligand Complex Preparation

Two crystal structures of the human glucokinase in complex with activators were downloaded from the RCSB PDB databank (Berman, 2000), with PDB IDs 1v4s (Kamata et al., 2004) and 4no7 (Petit et al., 2011). The sequences of the proteins were aligned and the amino acid subsequently renumbered, using the RCSB PDB comparison tool (Prlić et al., 2010) and the jFATCAT_flexible algorithm (Ye and Godzik, 2003). Amino acids 92–99 were not present in the 4no7 complex. Since they did not impact the protein stability during the simulations and no interactions with the ligand in the 1v4s system could be observed, they were not modeled. A table containing the alignment block is available in Supplementary Figure 1. The Maestro software (Schrodinger, 2010) was used to remove water moleculesp, add hydrogens and minimize the structures. The capping of the termini, the solvation and the addition of ions for the protein complexes had been set up through the CHARM-GUI web interface (Jo et al., 2008). Information about the prepared protein-ligand complexes is available in Supplementary Table 1.

### Molecular Dynamics Simulations

MD simulations were carried out using Amber 16 (Case et al., 2016). Parameters for the ligands were generated by tleap using the general AMBER force field (GAFF) (Wang et al., 2004). The MD simulation protocol started with an equilibration and thermalization phase of 125 ps with a 1 fs time step. Then each system was simulated for a total of 300 ns composed of 3 replicates of 100 ns with different initial velocities and using Langevin dynamics at a temperature of 303.15 K. The pressure was kept around 1 atm by a Monte Carlo barostat. The SHAKE algorithm (Ryckaert et al., 1977) was used to keep all bonds involving hydrogen atoms rigid. The time step of the production runs was set to 2 fs. Plots of the root-mean-square deviations for the proteins and their ligands are shown in Supplementary Figure 2.

### Library Generation

Compounds with experimental activities measured on human glucokinase were taken from the ChEMBL database (Gaulton et al., 2017). In total, 756 unique molecules with activity toward the target protein expressed in EC50 were extracted. This set was split based on the activity value threshold of 1.5 μM, resulting in 601 molecules labeled as actives and 155 as decoys. The KNIME Analytics platform (Berthold et al., 2009) was used in combination with the InteLigand Expert KNIME LigandScout Diversity Picker node (InteLigand Expert KNIME Extensions) to extract the 20,000 diverse molecules from the ChEMBL library based on extended connectivity fingerprint (ECFP) similarity, also labeled as decoys. Finally, a library for virtual screening was calculated using the idbgen algorithm in LigandScout 4.4 Expert (LigandScout 4.4 Expert). The procedure included the generation of a maximum of 25 conformations for each of the 20,756 molecules using the icon Fast settings (Poli et al., 2018). The active molecules were clustered in 5 groups based on ECFP similarity. Examples of the molecules present in each cluster is shown in Supplementary Table 2. The ligands from the x-ray derived structures PDB codes 1v4s and 4no7 were in cluster numbers 4, and 2, respectively.

### Pharmacophore Generation and Virtual Screening

Structure-based pharmacophore models were generated for each frame output from the MD simulations using LigandScout 4.4 Expert (Wolber and Langer, 2005). Models generated by LigandScout support the following chemical feature types: hydrophobic interactions, hydrogen bonds donor/acceptor, positive/negative ionizable area, aromatic ring and halogen bond donor features. In addition, pharmacophore models from the x-ray derived crystallographic structures of 1v4s and 4no7 were created. Water molecules were discarded before the generation of the models. The LigandScout activity profiling KNIME node was used to perform all virtual screening runs of models against the dedicated database of 20,756 molecules. Receiver operating characteristic (ROC) curves were generated for the virtual screening runs and the performance of the models was assessed by the calculation of area under the curve (AUC) values at specific percentages of the number of screened database molecules.

### Hierarchical Graph of Pharmacophore Models Generation

#### Feature Vectors and Graph Nodes

**Figure 1:**
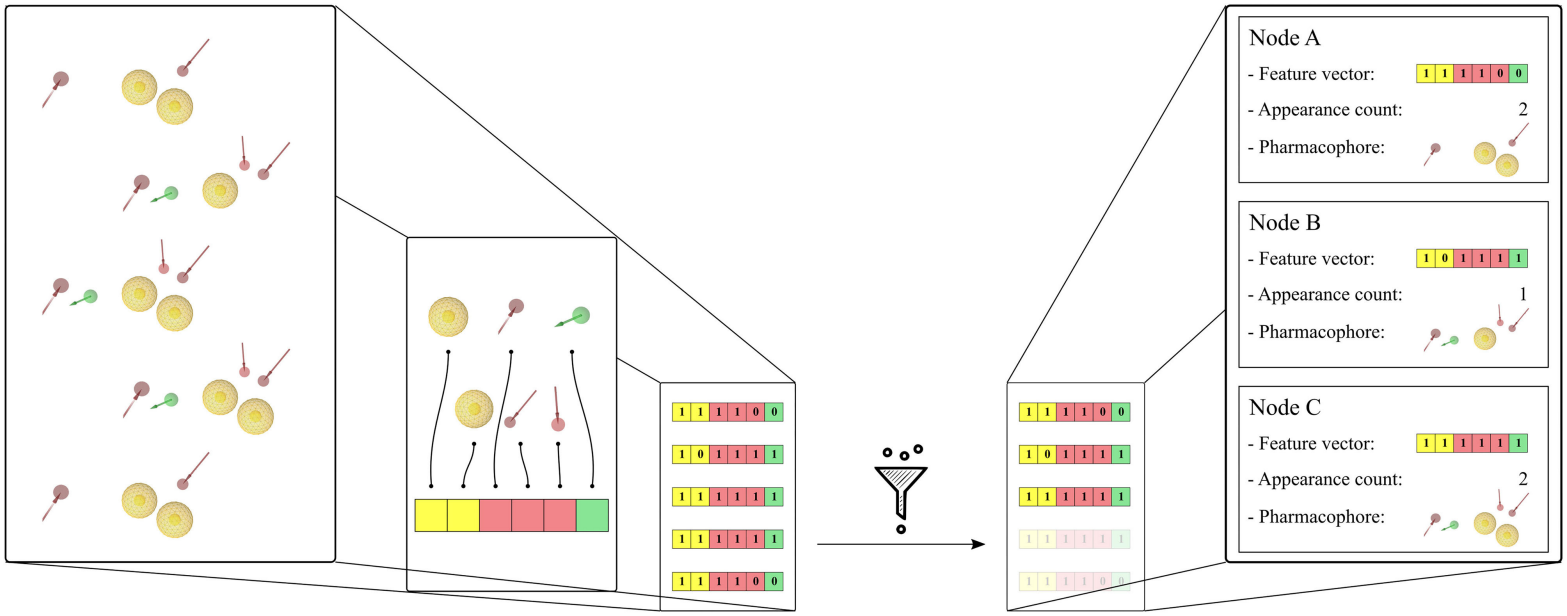
Generation of the feature vectors and their node representation from an initial set of 5 pharmacophore models. The set of pharmacophore models was converted into a corresponding set of feature vectors by first identifying all encountered unique features. Then the feature vector elements are initialized with 1 or 0 depending on the presence or absence of the corresponding unique feature in the pharmacophore model. A filtering step is done in order to remove duplicates with identical feature vectors. Finally, the graph nodes are created for each unique feature vector and the corresponding pharmacophore models and appearance count values are stored. The pharmacophore model features are: yellow spheres (hydrophobic), red and green arrows (hydrogen-bond acceptors and donors, respectively). The corresponding vector features are colored yellow, red, and green accordingly.

The pharmacophore models derived from the MD simulations were transformed into feature vectors in a related manner as described by the paper of Wieder et al. (2017). Each element of the vector represents a unique pharmacophore feature observed in the system. For this study, pharmacophore features are considered unique if they differ in any of the following components—pharmacophore feature type, ligand identifier, and/or identifier of the interacting environment residue(s). The 3D information is not taken into account in the identification of the unique pharmacophore features. Thus, e.g., a hydrogen bond acceptor feature generated for a ligand nitrogen atom that interacts with Serine will be considered as being different from a corresponding feature which represents the same nitrogen atom interacting with Threonine regardless of their shared feature type or their 3D position. Both pharmacophore features will be considered as being unique. In that, unique features are specific to the set of pharmacophores they were created from, as are the feature vectors. Feature vectors are represented as bit-strings describing the composition of the 3D pharmacophore models: a value of 0 simply denotes that the considered unique feature is absent in the model, and a value of 1 that the feature is present. A bit-string representation allows quick filtering of pharmacophore models with similar feature sets and furthermore enables a fast calculation of feature appearance counts during the simulation. Each node in the hierarchical graph representation is associated with a unique feature vector and contains additional derived information such as related frame number(s), appearance count, and linked pharmacophore models. Figure 1 shows the feature vector generation process for a set of pharmacophore models and their association to the graph nodes. To limit noise in the initial set of pharmacophore models, unique feature vectors are filtered according to their appearance count. The pharmacophore models were filtered to keep the models which appear at least 2 times as in the paper of Wieder et al. (2017), or 0.001 times the number of initial frames. The first pharmacophore models observed during the MD simulation for every unique feature vector in the hierarchical graphs were considered for VS.

#### Hierarchical Linkage

**Figure 2:**
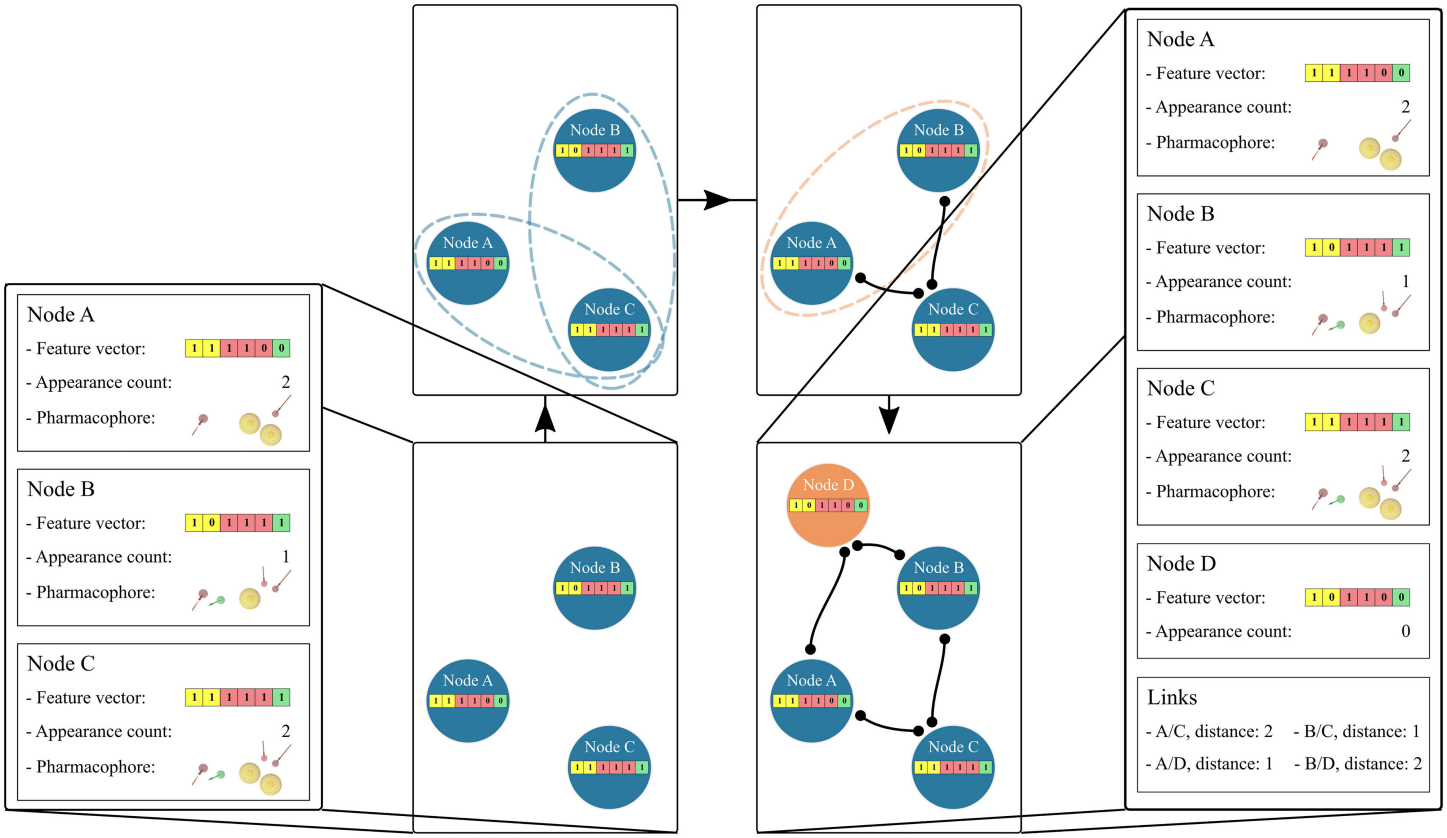
Hierarchical linkage of the graph nodes. Starting from 3 “Observed” nodes in blue, the stored feature vectors are tested for subset/superset relations, represented as blue dotted ellipses. Edges are then created if the relation is found. In the case that two nodes do not depict this relation, represented as an orange ellipse, a new node is created and linked to them. The appearance count for this “Artificial” node is set to 0 and its color is changed to orange. The pharmacophore model features are: yellow spheres (hydrophobic), red and green arrows (hydrogen-bond acceptors and donors, respectively). The corresponding vector features are colored yellow, red, and green accordingly.

The hierarchical linkage of the graph is based on the unique pharmacophore feature composition of the feature vector in each node. Links are created between nodes if their feature vectors are a subset or a superset of each other. Figure 2 depicts the linkage process. If two feature vectors do not exhibit a subset or superset relation, a new feature vector is temporarily created. This new feature vector represents the intersection set of the unique features for the two considered nodes. If this temporary feature vector is identical to an already existing node, the two considered nodes are linked to this one. If the temporary node is unique a new permanent node is created. The creation of “Artificial” feature vectors associated with a new node has been implemented to allow the generation of a unique hierarchical graph. Therefore, the “Observed” or “Artificial” nature of the feature vectors is stored as an attribute of each graph node in the form of its appearance count. Once all subset and superset links are generated, the redundant paths are removed.

#### Visualization

For an easy comprehension of the information contained in the graph nodes and their links, the visualization plays an important role. From the feature vectors, information about the composition of the pharmacophore models and the hierarchical links between them is already present. Several visual parameters can be used to depict additional graph properties. For this publication the following properties have been chosen, unless otherwise indicated:

- The appearance count of the pharmacophore models is represented by the size of the nodes. Therefore, the higher the appearance count, the larger visual representation of the node.
- The “Observed” or “Artificial” nature of the node is represented by its color. Blue is used to depict “Observed” nodes, and orange for “Artificial” nodes.
- The specificity of the pharmacophore models is represented by organizing the nodes in the x axis based on the number of unique pharmacophore features of which they are composed. Considering a node, each other node in the same column has the same number of pharmacophore features, each node on its left side is composed of fewer features and every node on its right is composed of more features.
- The representation of pharmacophore model similarity is achieved by dimension reduction using a Multidimensional Scaling Method (MDS) (Mead, 1992; Borg and Groenen, 2003). This method places all the elements of a distance matrix in a single dimension, preserving the distance between nodes as much as possible. The distance matrix is obtained by calculating the Manhattan distance between the feature vectors of the nodes. The similarity between the pharmacophore models is then represented by the relative distance between the nodes projected on the vertical axis of the graph. The reliability of this process is visualized by displaying the proportion of variance of the scaled data.

**Figure 3:**
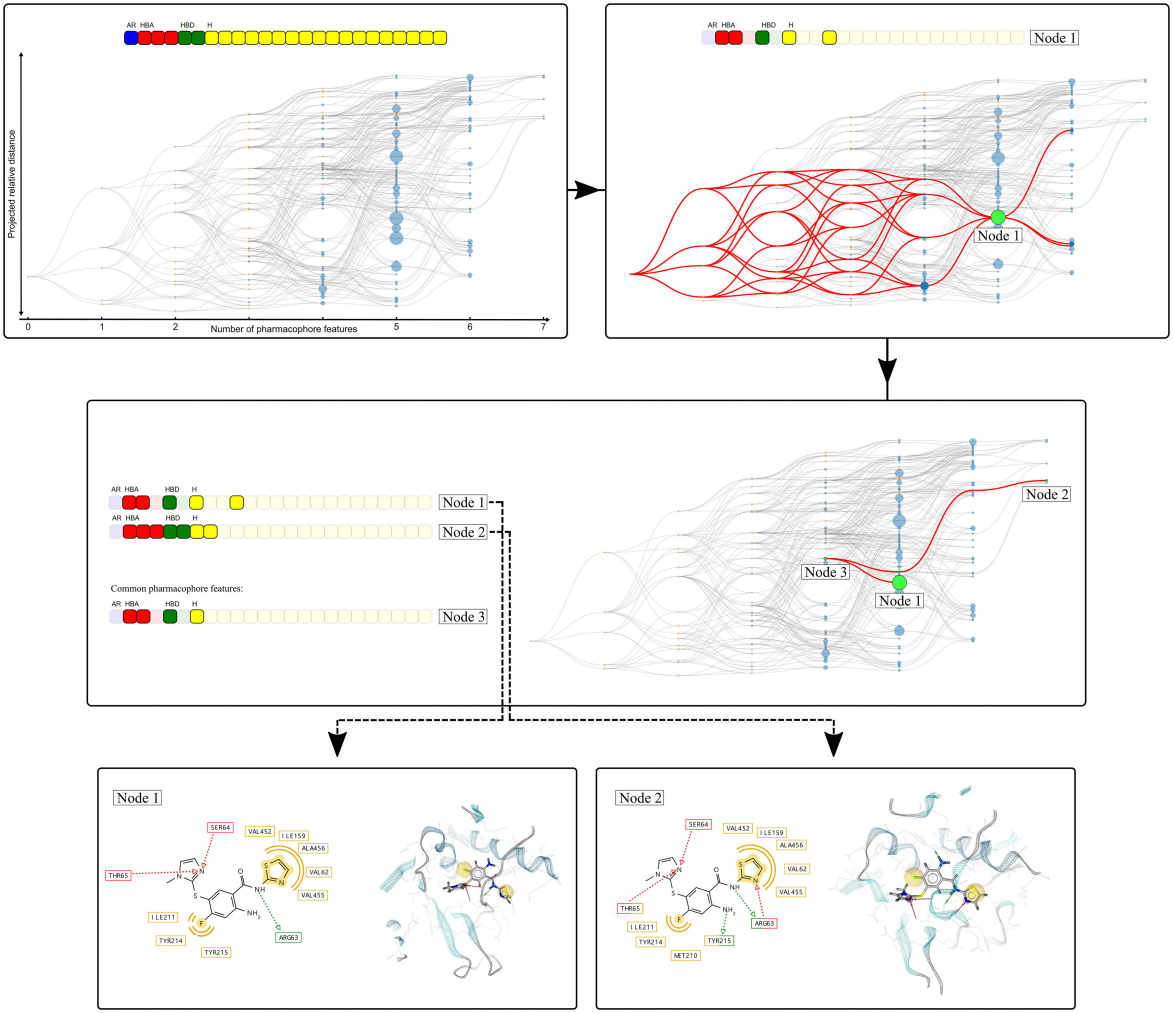
Visualization of the Hierarchical graph representation of pharmacophores models derived from a molecular dynamics simulation of human glucokinase in complex with an activator (PDB code: 1v4s). The feature vector is represented on the top of the graph and each box represents a unique pharmacophore feature. The color of the boxes indicates the type of the corresponding feature: yellow (hydrophobic), red and green (hydrogen-bond acceptors and donors, respectively), and blue (aromatic). The hierarchical graph below the feature vector represents all pharmacophore models observed during the simulation. Nodes are linked by hierarchical relations and their color denotes their origin: blue (“Observed” pharmacophore models), or orange (“Artificial” models only composed of a subset of features from the “Observed” pharmacophores). The graph is interactive, nodes can be selected to depict all related pharmacophore models, as shown with the selection of node 1. When two nodes are selected, the node that depicts the pharmacophore feature intersection set is also highlighted (Node 3), as depicted with the selection of Nodes 1 and 2. For each node, the associated pharmacophore model can be easily retrieved.

Figure 3 depicts an example of the graph representation.

## Results and Discussion

### Case Study: Glucokinase

The hexokinase IV, or glucokinase (GK) is an isoenzyme responsible for glucose phosphorylation (Beck and Miller, 2013). The concentration of glucose in the plasma determines the conformational switch of GK between its active and inactive states. The glucose level impact on GK activity makes this enzyme act as a sensor responsible for the glucose homeostasis in the human body (Bell and Polonsky, 2001). Therefore, GK has been a primary target for the development of antidiabetic drugs (Kamata et al., 2004; Osbak et al., 2009; Petit et al., 2011).

**Figure 4:**
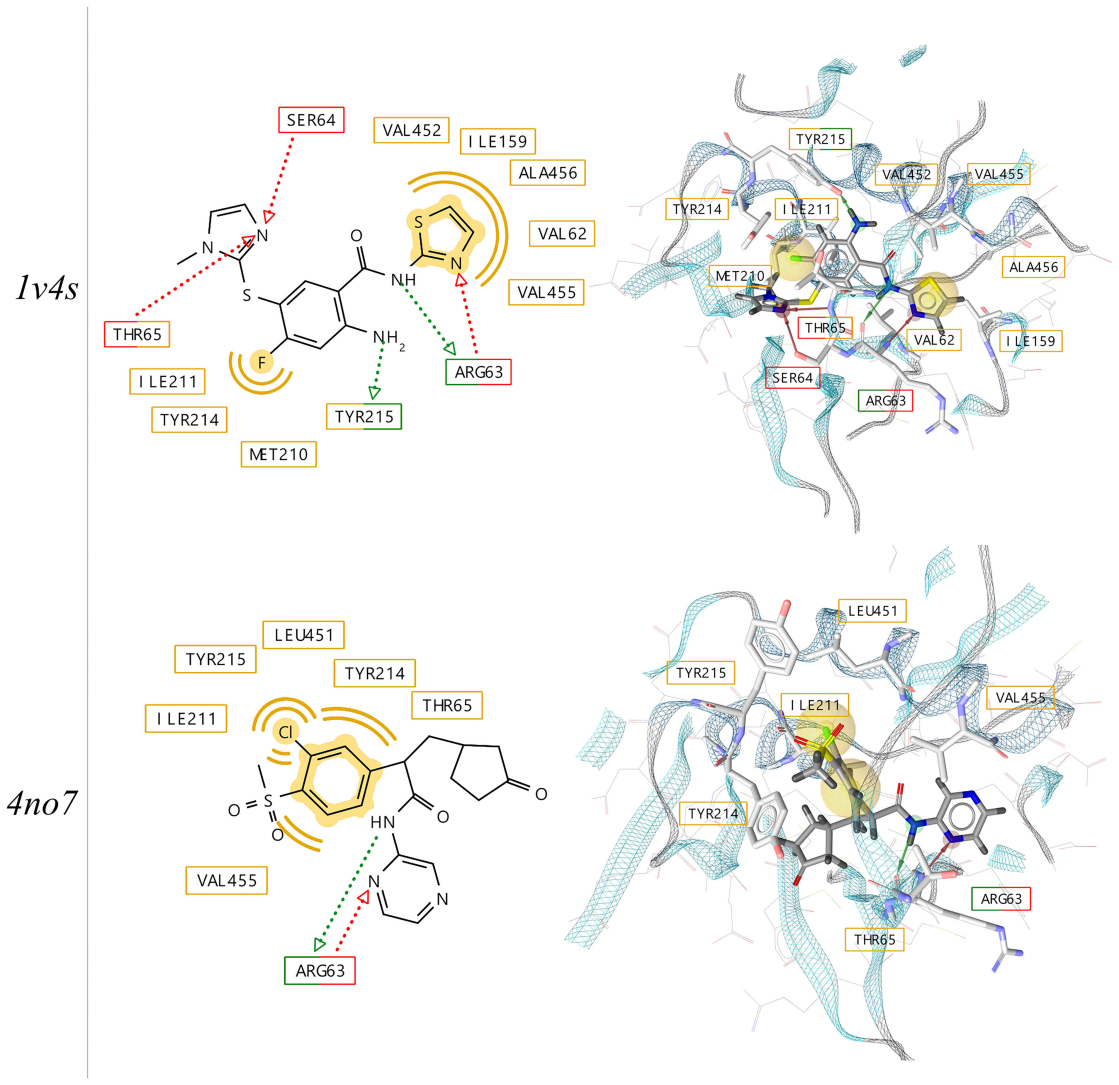
2D- and 3D-depictions of co-crystallized ligands and their putative interactions with human glucokinase derived from x-ray structures (PDB codes: 1v4s and 4no7) using LigandScout 4.4. Hydrophobic, hydrogen-bond donor and acceptor interactions are displayed in yellow (spheres), red and green (arrows), respectively.

Two crystallographic structures of the active conformation of GK with bound activators have been selected for this study (PDB codes: 1v4s and 4no7). The structures of the ligands, their position in the binding pocket and the pharmacophore features derived from the independent x-ray experiments are depicted in Figure 4. The binding poses of the two ligands show similarities with respect to pharmacophoric hydrogen bond donor and acceptor features capable of forming interactions with the backbone of Arginine 63. As it has been observed in previous studies (Petit et al., 2011), the loop comprising the residues 92–102 is poorly ordered, which results in the opening of an allosteric sub-pocket that can accommodate the chloro-phenyl-methane-sulfonate of the ligand in the 4no7 protein data bank (PDB) structure

### Hierarchical Graphs of Pharmacophore Models

Three MD simulation runs each of 100 ns were performed for both protein-ligand complexes. From the MD simulation trajectories obtained, 10,000 frames were extracted and subsequently used for the generation of pharmacophore models as described in the Methods part. The hierarchical graphs were then generated from the frame-based pharmacophore models including also the crystallographic structure-based pharmacophore models. The pharmacophore models were filtered according to their appearance count before subjecting them to the hierarchical graph generation procedure. The graphs were generated for each individual run as well as for the reunification of all runs for each crystallographic structure. Table 1 summarizes the composition of the graphs. In a previous publication (Wieder et al., 2017), we used a filtering criteria on the unique pharmacophore model appearance count in order to reduce the noise by removing pharmacophore models which appeared only once during the simulation. In this study, we investigated the impact of several values for the filtering criteria, discarding models appearing <2 frames up to <1% of the number of frames, in order to both reduce the noise and improve the readability of the graph. Source code for the generation and visualization of the graphs is available online (Source code for the HGPM Implementation, 2020). Source code for the generation and processing of LigandScout pharmacophore models has been excluded due to intellectual property reasons. Output data for an interactive demonstration of the Hierarchical Graph representation of Pharmacophore Models generated from the first MD run of 4no7 can also be accessed online (Demonstration of HGPM, 2020). A listing of all unique features for this system can be seen in the online demonstration when hovering the mouse over the feature vector.

**Table 1:** Results from the HGPMs obtained for each MD simulation providing the graphs node composition, pharmacophore filtering criterion, variance of the MDS projection, and the generation time.

**System**	**Run(s)**	**Number of pharmacophore models**	**Minimum appearance count of the pharmacophore model**	**Number of unique features**	**Number of “observed” nodes**	**Number of “artificial” nodes**	**Total number of nodes**	**Variance of the projection (%)**	**Time to generate (s)**
1v4s	1	10,001	2	63	515	478	993	11.5	30
			10	24	135	100	235	15.1	19
	2		2	56	457	464	921	12.5	26
			10	27	145	119	264	15.5	19
	3		2	52	549	766	1,315	13.4	50
			10	28	160	243	403	14.5	20
	1,2,3	30,001	2	81	1,163	1,207	2,370	11.7	258
			10	41	346	337	683	13.3	54
			30	28	174	186	360	14.9	51
4no7	1	10,001	2	37	814	680	1,494	12.7	63
			10	26	177	139	316	17.1	18
	2		2	36	813	645	1,458	10.7	51
			10	23	172	133	305	14.0	17
	3		2	39	852	728	1,580	10.2	60
			10	25	191	150	341	13.4	21
	1,2,3	30,001	2	50	1,807	1,394	3,201	10.3	511
			10	29	461	285	746	13.5	51
			30	26	172	130	302	16.2	50

### Analysis of the Unique Feature Vectors

**Figure 5:**
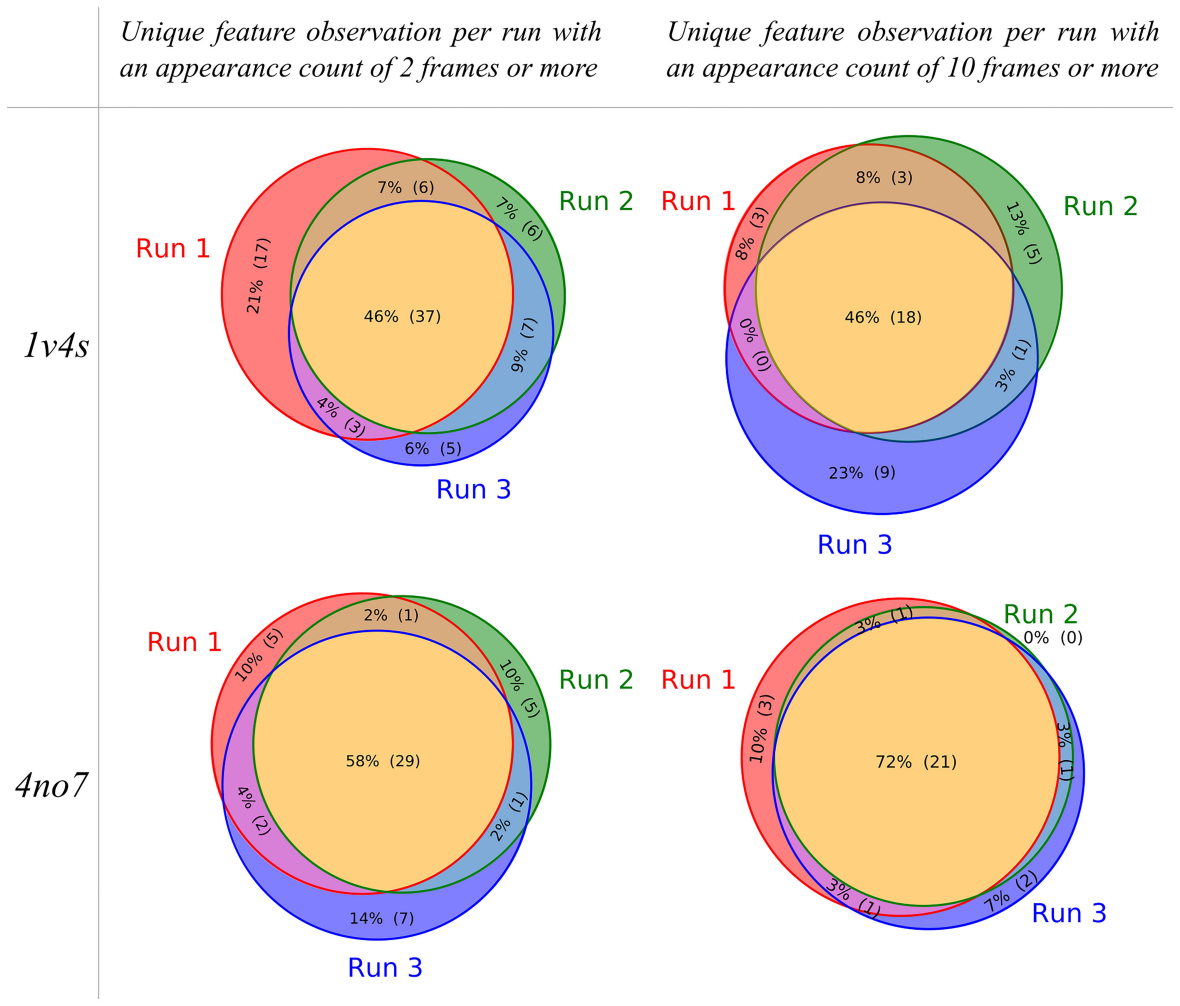
Venn diagrams showing the unique pharmacophore feature similarities between the MD simulation runs performed for the 1v4s and 4no7 systems, respectively. The pharmacophore models considered for the sampling of the unique features were filtered based on an appearance count criteria, set to 2 for the diagrams on the left and 10 on the right.

To visualize the composition of the pharmacophore models that were obtained for each performed MD simulation of the systems, the individual partitioning of the unique observed features is represented as Venn diagrams shown in Figure 5. For the 1v4s system, 37 out of 81 (46%) of all unique observed features are present in every of the 3 MDs runs if the appearance count filtering criteria is set to 2 frames or more. This ratio stays similar when the appearance count criteria is set to 10 frames or more, resulting in 18 out of 39 (46%) unique observed features being in common. Although approximately half of the unique features are always observed, each individual run led to the observation of 5–17 unique features which are only present in this specific run (filtering criteria of 2 frames or more). When the filtering criteria is set up to 10, the number of unique features solely observed in a specific run decreases, but the ratio stays similar.

Considering the 4no7 system, the trend slightly differs with an increase in the ratio of unique features in common to the 3 runs. We observed 29 out of 50 (58%) unique common features with the appearance count filtering criteria set to 2 frames or more and 21 out of 29 (72%) with the filtering criteria set to 10 or more. Consequently, the number of unique features solely observed in single runs decreased between 5 and 7 with the filtering criteria set to 2 and 0–3 with the criteria set to 10. While several unique features were always present regardless of the run, each individual MD simulation provided exclusive information. Every further analysis presented in this work was made considering every pharmacophore model obtained from any of the 3 runs performed for each system.

**Figure 6:**
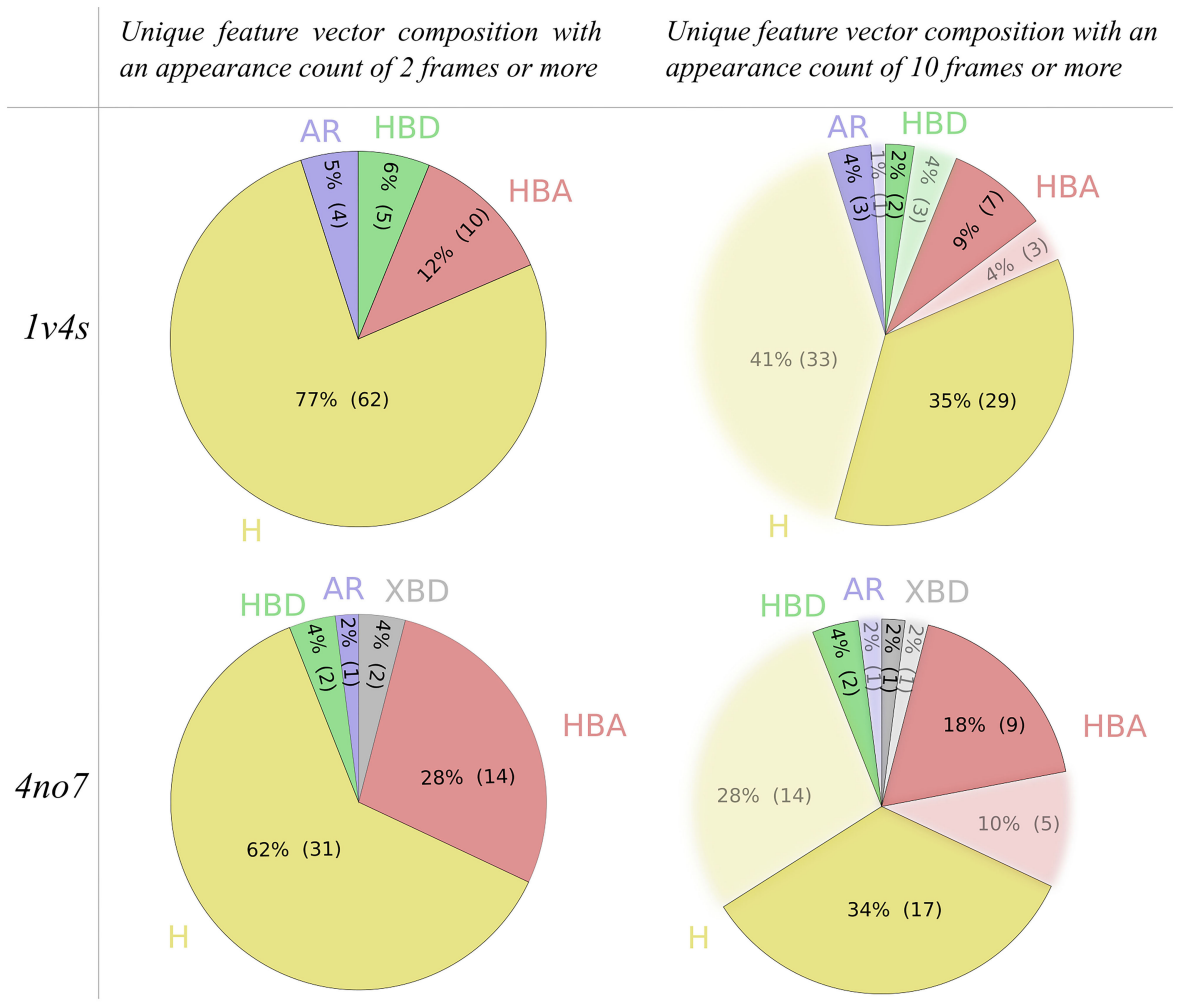
Pie charts of the unique pharmacophore feature composition of the feature vectors generated from all 3 MD simulations, based on the applied appearance count filtering criterion for both the 1v4s and 4no7 systems. Hydrophobic interactions are colored yellow, aromatic interactions blue, hydrogen bond donors green, hydrogen bond acceptors red and the halogen bond donors gray.

The filtering criteria applied on the appearance count have been set to decrease the complexity of the hierarchical graphs in terms of number of nodes and links, as can be seen in Table 1. To investigate the impact of the filtering criterion on the feature vector composition, Figure 6 depicts details of the nature of the unique pharmacophore features. Among all unique pharmacophore features observed in the MD simulations for 1v4s and 4no7 62 out of 81 and 31 out of 50 are hydrophobic interactions. This high number of hydrophobic features is partly caused by the nature of the feature serial generation algorithm. For example, a hydrophobic feature that involves the fluorine atom of the ligand in 1v4s and amino acids ILE211, TYR214, TYR215 will be considered different from another hydrophobic feature involving the same fluorine atom on the ligand, but different amino acids like THR65, MET210, ILE211, TYR214, TYR215, even though every amino acid of the first feature is also involved in the second one. This is inherent in the LigandScout definition of hydrophobic features and has been kept as is.

However, setting the appearance count filtering criterion to 10 frames or more tends to discard more hydrophobic features than any other type of pharmacophore interaction, as it can be observed for both systems shown in Figure 6. Table 1 additionally provides information about hierarchical graphs with an appearance count set to 1% of the number of frames. For graph readability reasons, we chose to use a filtering criterion of 10 for the rest of this study.

### Uses and Analysis of the Hierarchical Graphs

**Figure 7:**
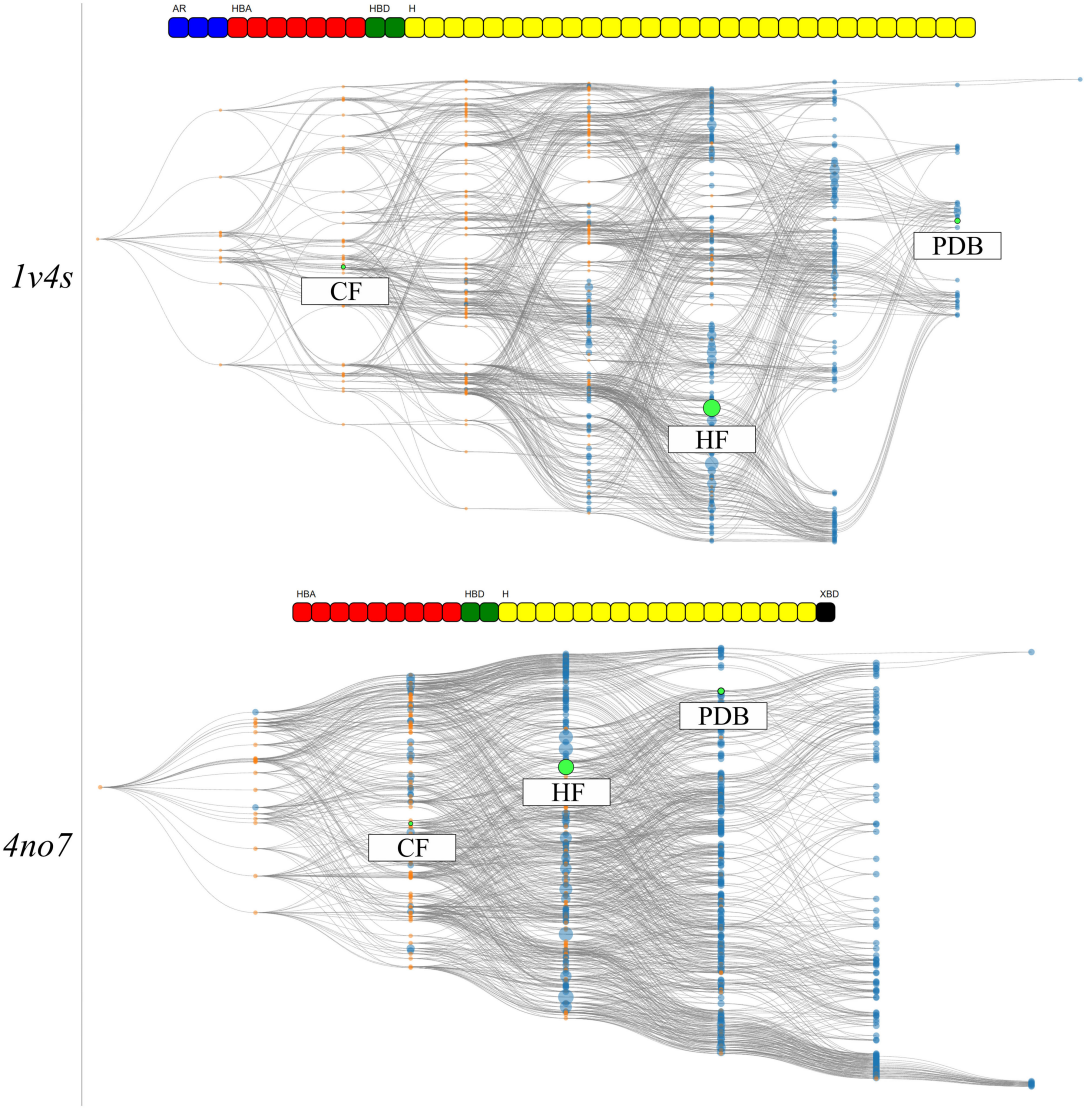
The HGPMs based on the 3 MD simulation runs of the 1v4s system is shown on the top, and for the 4no7 system on the bottom. The nodes matching the pharmacophore models obtained from the PDB structure, the models with the highest frequency of appearance and with the highest count of common features between the crystallographic structures of the systems are highlighted in green and labeled HF and CF, respectively. The feature vectors of the highlighted models are shown above of the corresponding hierarchical graphs.

The hierarchical graphs of both investigated systems are shown in Figure 7. A detailed description of the information which can be visually retrieved from these graphs is given in detail in section Visualization.

For system 1v4s, the graph spans nine columns and thus includes models with up to 8 pharmacophore features. Several nodes are converging close to the pharmacophore model obtained from the crystallographic structure, labeled as PDB. However, no other pharmacophore models were observed that comprise a superset of the features represented by the PDB node. The node with the highest appearance count (HF node) among the 3 runs is vertically displaced from the PDB node and while they share 3 features, they are not directly related in terms of feature hierarchy as shown in more detail in Supplementary Figure 3. The node associated with the hydrogen bond donor and acceptor features that interact with Arginine 63 (CF node) is located on the third column of the graph, since it possesses a low number of features and is therefore not specialized. The PDB and HF nodes were used to perform VS runs against the active and decoy database as described in the Methods section. In addition, a selection of all pharmacophore models which represent a superset of the HF and CF nodes were used for a consensus screening run. The CHA (Wieder et al., 2017), that is a consensus approach considering all observed pharmacophores was also carried out. A summary of the VS results can be found in Table 2.

**Table 2:** Virtual screening results for both systems using different selections of pharmacophore models.

**System**	**Selection**	**Number of common features**	**Number of pharmacophore model(s)**	**Number of hits**	**Auc at 1%**	**Auc at 5%**	**Auc at 10%**	**Auc at 50%**	**Auc at 100%**
1v4s	pdb	7	1	0	0.00	0.00	0.00	0.00	0.00
	CHA	0	346	3,676	0.96	0.95	0.93	0.86	0.74
	HF	5	1	65	0.98	0.90	0.81	0.59	0.53
	HF+	5	7	87	0.98	0.91	0.82	0.59	0.53
	CF+	2	236	1,337	0.98	0.96	0.95	0.82	0.68
4no7	pdb	4	1	401	0.98	0.77	0.68	0.54	0.51
	pdb+	4	6	655	0.96	0.82	0.74	0.56	0.52
	CHA	0	461	13,868	0.98	0.79	0.71	0.64	0.58
	HF	3	1	2,761	0.81	0.41	0.34	0.51	0.50
	HF+	3	22	3,545	0.95	0.82	0.72	0.56	0.52
	CF+	2	49	2,546	0.93	0.81	0.74	0.59	0.54
	Selection +	3	30	3,421	0.96	0.88	0.81	0.58	0.53

*Selections labeled with a + represent a subset of models used for consensus scoring. Model subsets comprise every pharmacophore with at least the same features as the initial node*.

We observed that the PDB pharmacophore for 1v4s did not retrieve hits during the VS, which might be due to the number of defined and specific directional hydrogen bond vectors defining its specific binding mode, with 5 of the 7 pharmacophore features containing defined directions for hydrogen bonding. The 4 other pharmacophore selections for this system performed well, with area under the curve (AUC) values above 0.96 at 1% of the number of database molecules. The HF pharmacophore model performed as well as the other models regarding the AUC at 1% but slightly below for the other and retrieved 65 hits out of 20,756 molecules. The selection of all superset models of the CF node provided the most stable and best results regarding the AUC, even outperforming the CHA. Those good results can be linked to the high amount of pharmacophore models which were used for screening, 236 for the CF+ selection and 346 for the CHA. Lastly, the set of all pharmacophore models which are supersets of the HF nodes performed close to the HF node alone but retrieved 87 molecules.

The overall hierarchical graph of pharmacophore models of the 4no7 system differs from the one of the 1v4s system mainly by the two branches involving the nodes with the highest number of features on the right side of the graph. Additionally, the PDB, HF, and CF nodes are vertically quite close and are hierarchically linked as it can be seen in Supplementary Figure 4. This leads to the conclusion that these three nodes are part of only one of the two branches among the most specialized pharmacophore models. Therefore, an additional node has been selected to involve an arbitrary model of the second branch, which has been labeled as “Selection” in Figure 7. Table 2 shows the VS results obtained with the PDB and HF nodes. The CHA was applied and additional consensus scoring runs were made considering the pharmacophore supersets of the PDB, HF, CF and custom selected nodes. For 4no7, the PDB pharmacophore obtained the best AUC results at 1% of the database and retrieved 401 molecules. The CHA performed as good as the PDB node regarding the AUC at 1% and showed its stability by delivering comparable AUC values at higher percentages than the other approaches while retrieving 13,868 molecules. The HF node model did not perform as well as other models with an AUC value of 0.81 at 1% that fell to 0.41 at 5%. The selection of all superset models of the HF node, however, achieved significantly better VS results than the HF node alone. Lastly, the selection based on the “Arbitrary” node delivered high AUC values with the best observed results at all thresholds superior to 1%. It is interesting to point out that the “Arbitrary” selected node and the HF node are of disparate feature composition, but still deliver high AUC values. To scrutinize this observation, we looked at the structural clusters of the active molecules retrieved by these two approaches, as detailed in Supplementary Figure 5. We observed that for a hitlist truncated at 2,076 molecules (10% of the database), the HF node retrieves a consequent number of molecules from all 5 clusters, when the arbitrary selected node selection mostly retrieves molecules from the cluster numbers 2, 3, and 5. This difference in the virtual screening hitlist composition suggests the presence of two different binding modes, involving structurally distinct active molecules. Therefore, investigating why these two different branches are nonetheless able to distinguish between active and inactive molecules can be of high value for elucidating the binding modes of highly affinity GK ligands. It can also be highlighted that without prior knowledge of the systems, the selections of multiple pharmacophore models proved to be especially stable and reliable in terms of AUC values in comparison to the single pharmacophore models.

### Analysis of Hierarchical Graphs of Pharmacophore Models Colored by Virtual Screening Results

**Figure 8:**
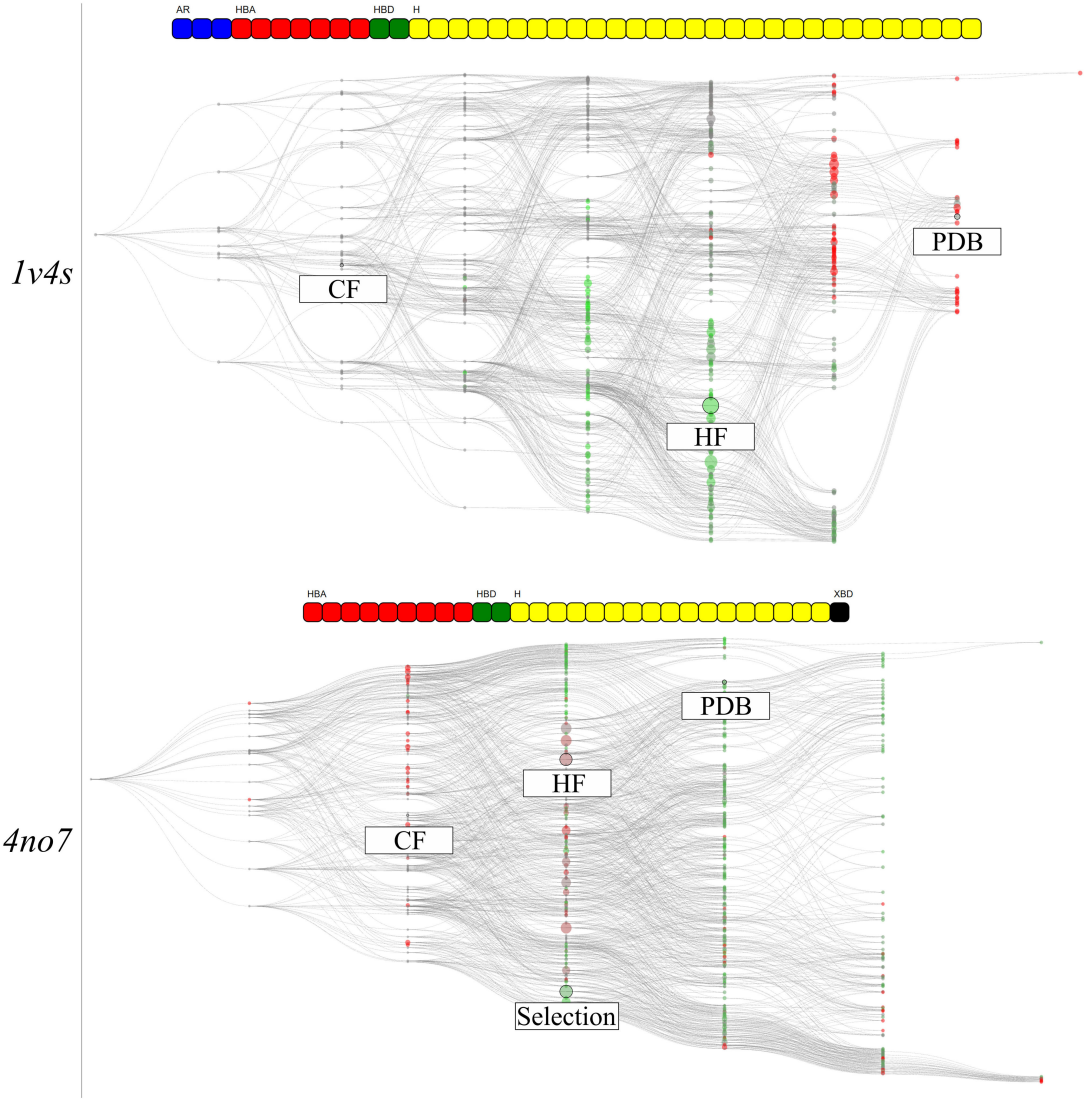
The hierarchical graph of pharmacophore models derived from human glucokinase PDB codes of the 1v4s system is shown on the top, and of the 4no7 system on the bottom. The nodes are colored according to their AUC values at 10% of the database molecules. The greener the node, the closer is its AUC value to 1, and the redder the closer to 0. Nodes with “Artificial” pharmacophore models are colored gray. The nodes corresponding to the pharmacophore models obtained from the PDB structure, from the highest frequency of appearance and with the two common features between the systems are labeled accordingly. Associated feature vectors are shown above the corresponding graphs.

The hierarchical graph representations provided intuitive support for the selection and evaluation of pharmacophore models, as detailed in the previous part. Nonetheless, additional information can be depicted to emphasize special characteristics of the pharmacophore models. To better understand the features involved in correctly distinguishing active and inactive molecules, all pharmacophore models associated with the feature vectors of the hierarchical graphs were used for VS. Then, each node was colored according to its AUC value at 10% of the database. For both systems, the corresponding results are shown in Figure 8. In the shown hierarchical graphs, the greener the node the closer its AUC value is to 1, and the redder its color becomes, the closer its AUC value is to 0. The “Artificial” nodes were not used for VS runs and are colored gray.

The graph of the 1v4s system shows a clear separation of colors. All nodes in the area of the HF node, as well as all its superset nodes are depicted in green. Therefore, prioritizing a selection of pharmacophore models from this area might be indicated in order to find the best balance between consensus virtual screening performance and number of considered models. On the other hand, the top half of the graph mainly depicts bad performing models, including the PDB node. The region around the CF node is depicted in gray as the VS was not performed for the pharmacophore model of the “Artificial” nodes. However, the consensus VS runs using all pharmacophore model supersets of the CF nodes performs well, although the sets contain both individual good and poorly performing models.

The graph for the 4no7 system is homogeneous and most of the nodes deliver good VS results (AUC > 0.5). The HF node is colored brown, leading to below average results. However, the more specialized nodes located on the top branch of the graph performed well, including the PDB node. The region around the custom selected node, present on the other branch, also performed well, which can be interpreted as a different ligand binding mode. All models from the “Observed” nodes in the same columns as the CF node are displayed in red since models with less than three features do not lead to an unambiguous model alignment in 3D space and therefore return no hits.

### Analysis of the Hierarchical Graph of Pharmacophore Models Projected to the GK Protein

**Figure 9:**
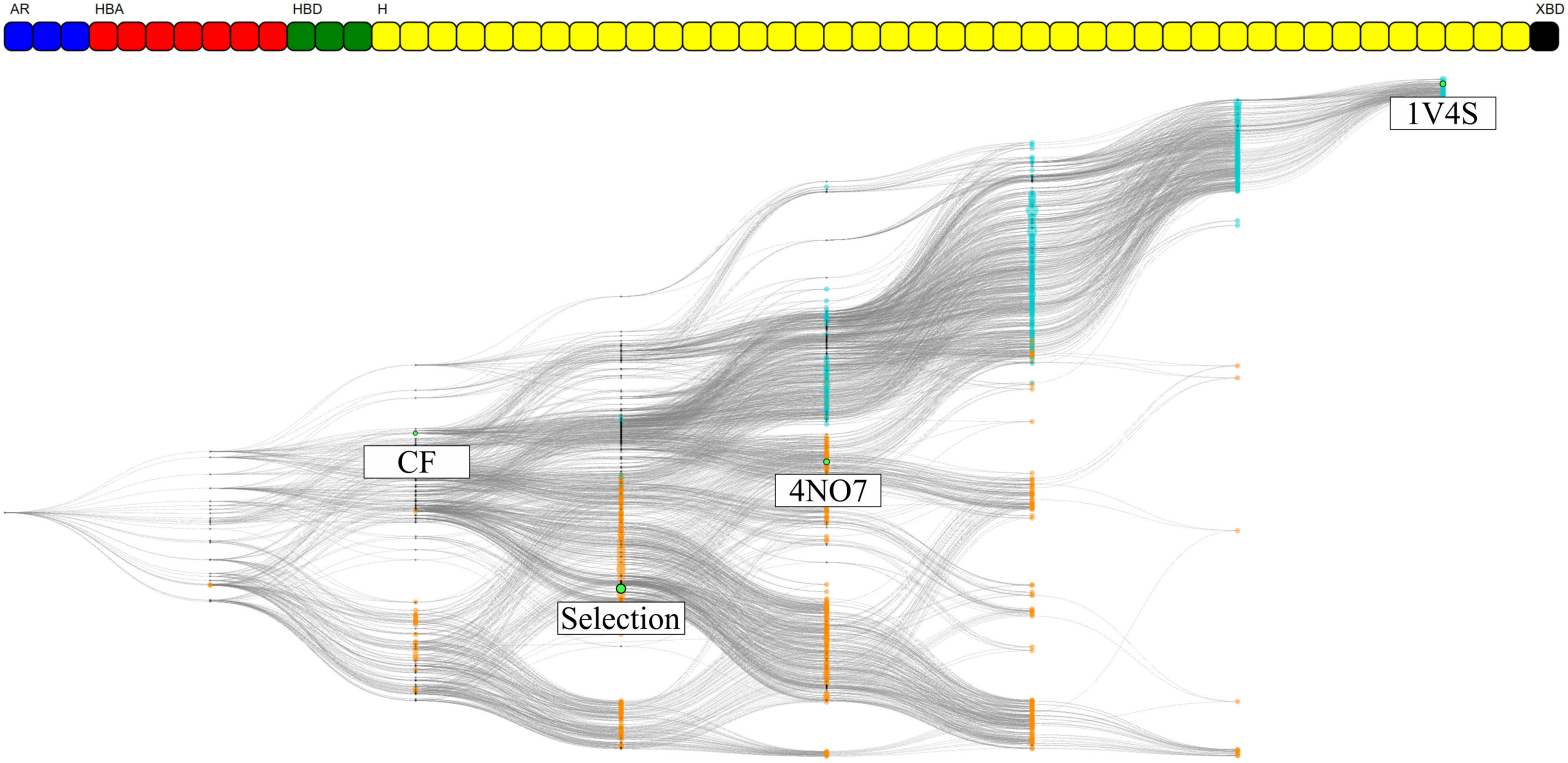
HGPM obtained from the MD simulation runs of both the 1v4s and 4no7 system. The nodes are colored based on the system they were derived from: cyan for 1v4s and orange for 4no7. The nodes matching the pharmacophore models obtained from the PDB structures, the custom selection and the node with the two observed common features of the systems are labeled and highlighted in green. The feature vector is displayed on the top of the graph.

The information gained for each individual system is present in the form of pharmacophore selection and in the comparison of the feature composition of the nodes. This is due to the pharmacophore feature label generation algorithm that considers which part of the ligand is interacting and allows a better accuracy in distinguishing unique features. Thus, the comparison between two systems with different ligands, as we did it for the 1v4s and 4no7 crystallographic structures is not possible. However, by disregarding the ligand identifier in the pharmacophore feature serial generation procedure we only keep the type of interaction of the pharmacophore feature and the protein identifier. In this way, we are trading the accuracy of where the interaction occurs on the ligand side, for the ability to ‘project’ the interaction on the protein side. This therefore unlocks the possibility to consider all pharmacophore models generated from a specific protein, regardless of which ligand was involved in the pharmacophore interactions. To demonstrate this alternative way of generating the pharmacophore feature serial, a HGPM based on all runs from both 1v4s and 4no7 systems has been created and is shown in Figure 9. Detailed information about the graph is provided in Supplementary Table 3.

The number of graph nodes and the number of unique pharmacophore features present in this hierarchical graph are both greater than those of the 2 previous graphs, which increases the complexity of the depiction. Among the 55 unique observed features, 7 where shared between 1v4s and 4no7: 3 hydrogen bond acceptor interactions with Arginine 63, Serine 64 and Threonine 65; a hydrogen bond donor interaction with Arginine 63; 3 hydrophobic features engaged in interactions with Tyrosine 24, the amino-acids Isoleucine 211, Threonine 65 and Tyrosine 214 and the amino-acids Threonine 65 and Tyrosine 214. Despite these common interactions, no “Observed” pharmacophore models were present in both the 1v4s and 4no7 MD simulation runs. We can nonetheless clearly observe a distinction between the two colored clusters, depicting the initial node affiliation. The top half of the graph with cyan colored nodes represent all pharmacophore models from the 1v4s system, where we can recognize a single branch leading to the specialized pharmacophore model of the PDB structure. For the bottom half of the graph with the orange nodes from the 4no7 system, we observe a more homogeneous spacing. The two branches previously observed are not easily distinguishable. It can be pointed out that the 4no7 PDB node is located near the nodes originating from 1v4s, and the custom selection node tends to fork more to the bottom part of the graph as more detailed on Supplementary Figure 6. As the feature vector differs from the individual graphs, the pharmacophore selection for the CHA and for the subsets of pharmacophore models with the same feature as the CF nodes were different than previously and their virtual screening results details for this graph are presented on Supplementary Table 4.

**Figure 10:**
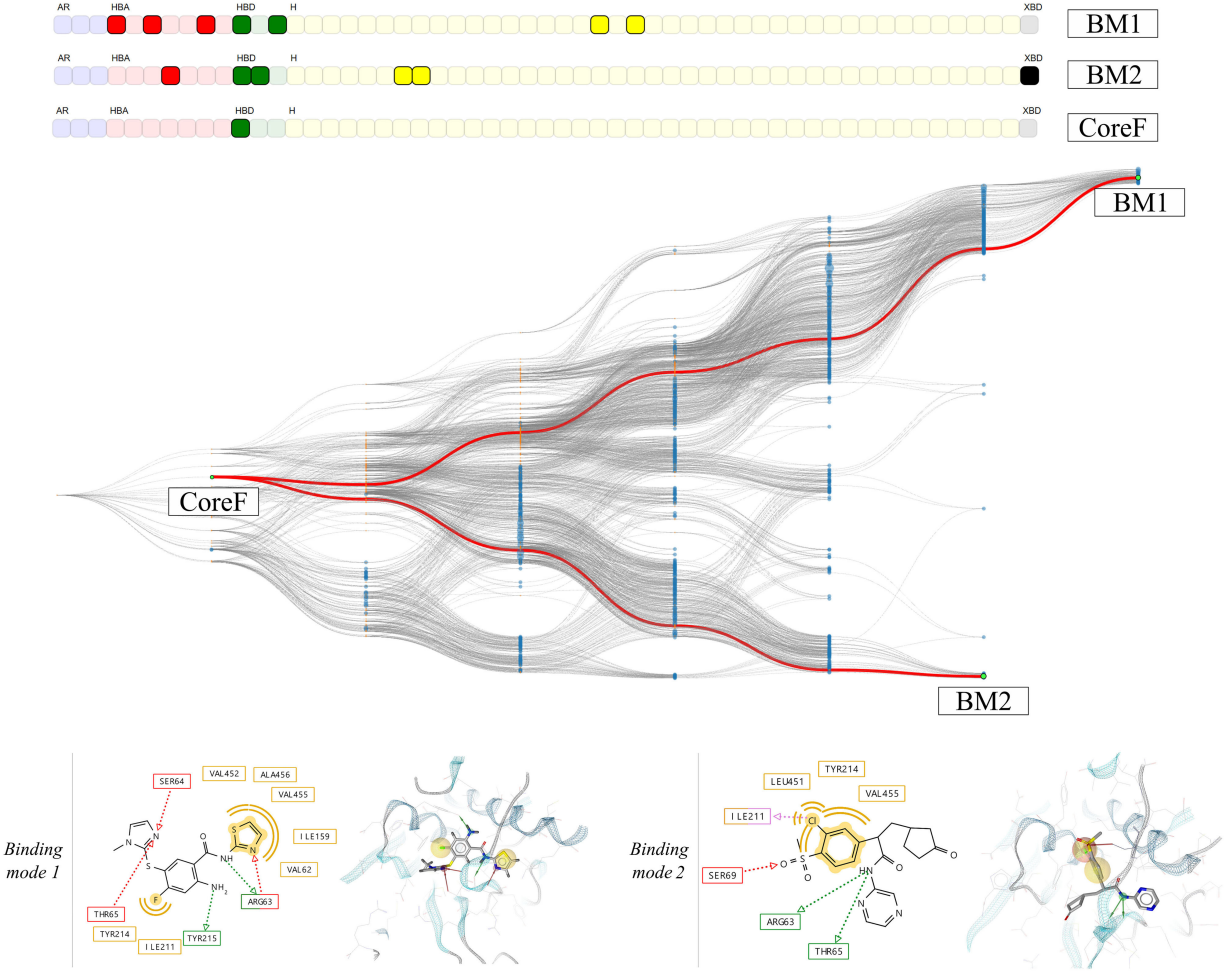
HGPM obtained from the MD simulation runs human glucokinase starting from both the 1v4s and 4no7 systems. The nodes BM1 and BM2 were selected to show examples of different observed binding modes. 2D and 3D depictions of the corresponding pharmacophore models are shown below the graph.

Based on the heterogeneity of the graph, two pharmacophore models were selected. The hierarchical graph as well as two selected nodes, respectively binding mode 1 (BM1) and 2 (BM2) are shown in Figure 10. Both models are among the most specialized observed pharmacophores with 7 and 6 features, respectively. The single shared feature is a hydrogen bond acceptor interaction with the Arginine 63, whose corresponding node is labeled CoreF. In the 3D representations of the pharmacophore models in Figure 10, we observed that the hydrophobic and halogen bonding interactions are present in different areas of the GK pocket as they involve different amino acids. Due to the important difference in the pharmacophore models in terms of feature composition and 3D alignment, the observed results can be linked to distinct binding modes due to the presence of the allosteric sub-pocket. This information can be used to provide a better depiction of the specific binding modes than the initial comparison between the two PDB pharmacophore models. Additionally, it allows the selection of smaller pharmacophore sets for consensus virtual screening in comparison with the CHA or the CF+ selection of models.

## Conclusion

Motivated by the recent interest in consensus-based virtual screening methods involving pharmacophore models (Wieder et al., 2017; Polishchuk et al., 2019; Madzhidov et al., 2020), we developed an intuitive hierarchical graph representation of pharmacophore models. A user-friendly interactive visualization of the pharmacophore-based graph provides valuable information for computational chemists toward the understanding of protein-ligand interaction patterns and can aid in the selection of pharmacophore models for VS experiments. The graph can be created from sets of pharmacophore models generated for multiple crystallographic structures with identical macromolecular targets or for the output of MD simulation runs to provide insight into the dynamic aspects of the investigated systems. The graph generation has proven to be computationally inexpensive as it takes seconds to be created even for bigger ensembles with more than 10,000 models (see Table 1).

We selected two crystallographic structures of the human glucokinase to evaluate the HGPM generation algorithm in its ability to identify different binding modes and to select small representative pharmacophore model sets for consensus VS experiments. MD simulations and graph generations were performed individually for the two systems. Different selections of pharmacophore models were used to distinguish between active and inactive molecules for the two investigated systems. The selection of all models which possess a superset of the features contained in the pharmacophore model with the highest appearance count performed similarly to the CHA method in terms of AUC value and stability, while at the same time reducing the number of considered models for the 1v4s system by 20-fold and by more than 45-fold in the case of the 4no7 system (see Table 2), and thus helped to significantly reduce the required screening time. The hierarchical graph in Figure 8 also helped to identify the best performing ensembles of pharmacophore features by depicting the virtual screening results for every pharmacophore model. Although the presence of two specialized branches in the hierarchical graph of the 4no7 system (in Figure 7) has already been noticed, the two different binding modes of GK were clearly identified by the hierarchical graph generated from the models extracted from both the 1v4s and 4no7 MD simulations (Figure 10), which is in perfect agreement with literature (Petit et al., 2011).

Depending on the goal, the graph representation can be adapted flexibly by either changing the pharmacophore feature serial generation algorithm or by showing additional properties. Highlighting the hierarchical relationship between pharmacophore models, this graph allows the user to analyze a target system by comparing the composition of several pharmacophore models in a single graphical representation, thus promoting the understanding of the binding process and the selection of pharmacophore models for consensus virtual screening runs. A typical workflow using the information provided by the graph representation is e.g., to first select a single pharmacophore model with the highest observed frequency, then perform a virtual screening run and finally add or remove individual features identified by following hierarchical links to build a refined model with the best ratio between accuracy and specificity. Presenting the hierarchical graph of pharmacophore models, we want to introduce an intuitive representation of multiple pharmacophore models and provide the computational and medicinal chemists with a new tool to enable an advanced understanding of the protein-ligand binding process, allowing for better decision support in the process of optimizing bio-active molecules.

## Data Availability Statement

The datasets presented in this study can be found in online repositories. The names of the repository/repositories and accession number(s) can be found at: http://www.wwpdb.org/, 1v4s http://www.wwpdb.org/, 4no7.

## Author Contributions

GA, BS, TI, DP, and LT: project conception. GA, ST, TI, and LT: methodology and project administration. GA, WO, BK, and IG: method implementation and validation. GA: writing and original draft preparation. WO, ST, BS, TI, DP, and LT: reviewing and editing. LT: supervision and funding resources. All authors contributed to the article and approved the submitted version.

## Conflict of Interest

BS and IG are employees of the company Inte:Ligand Software-Entwicklungs und Consulting GmbH, Vienna, Austria. TI and DP are employees of Institut de Recherches Servier (IdRS), Croissy-sur-Seine, France. The herein presented work is the result of a joint research project funded by the aforementioned companies. The remaining authors declare that the research was conducted in the absence of any commercial or financial relationships that could be construed as a potential conflict of interest.
