# Supplementary material for: Hierarchical Graph Representation of Pharmacophore Models
Source: Front Mol Biosci. 2020 Dec 14;7:599059. doi: 10.3389/fmolb.2020.599059 (PMC7793842; doi:10.3389/fmolb.2020.599059)
Supplement: Supplementary file 1 [file Data_Sheet_1.PDF]

Supplementary Material

1 Supplementary Figures and Tables

1.1 Supplementary Figures

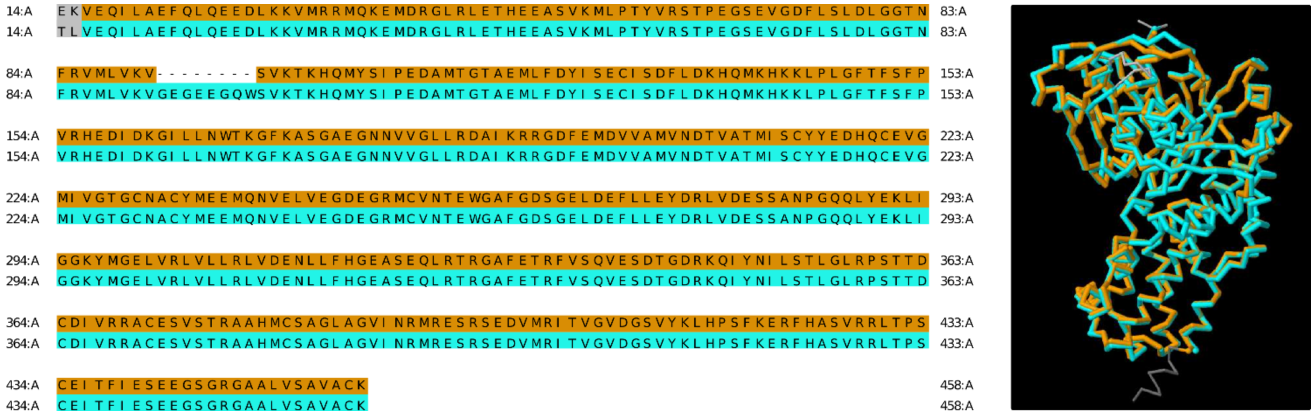

Supplementary Figure 1: Amino acid sequence alignment block for the crystallographic structures 1v4s in cyan and 4no7 in orange. The picture on the right shows the 3D alignment of the two proteins.

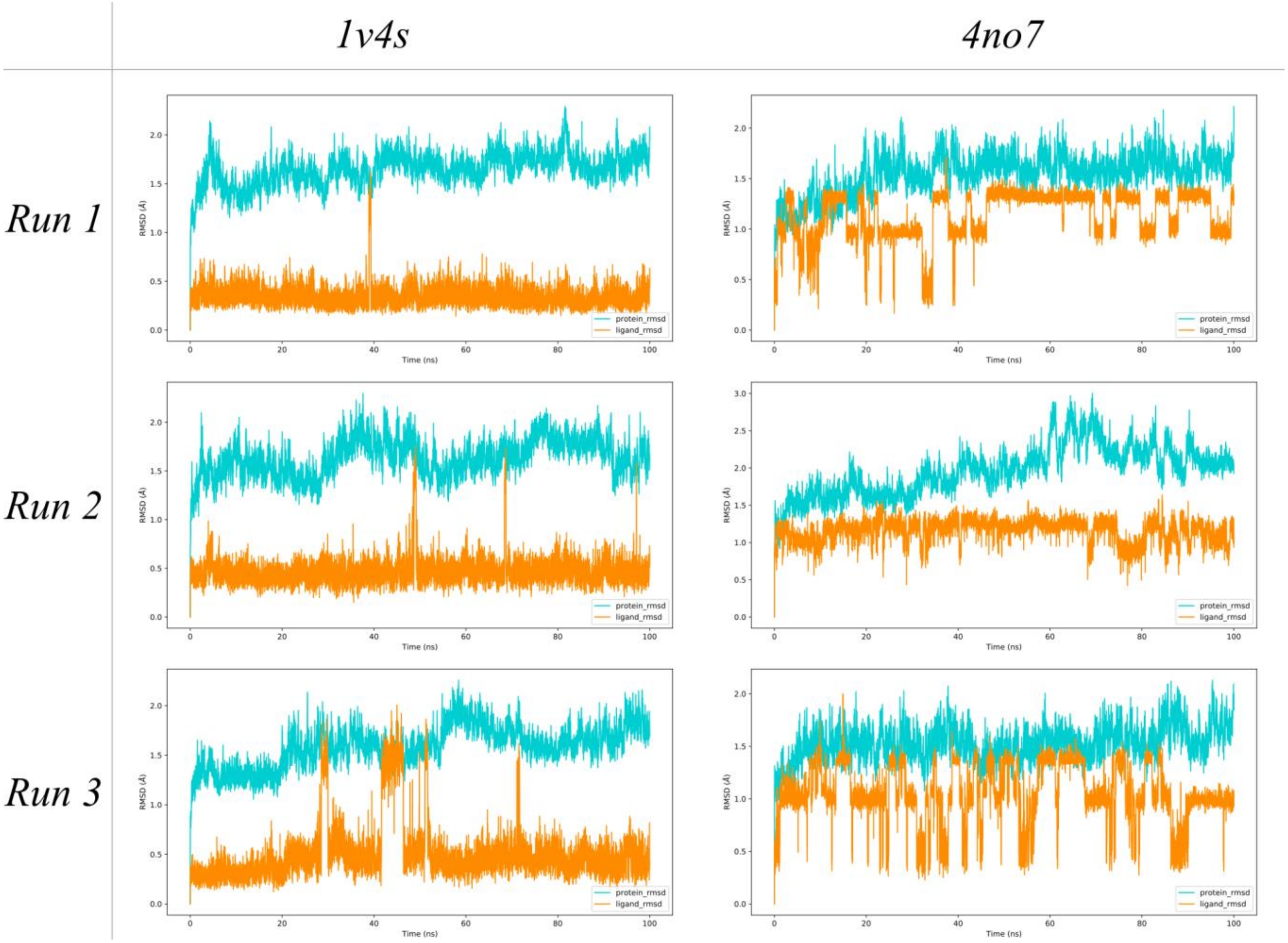

Supplementary Figure 2: Molecular dynamics simulations root-mean-square deviations for the two protein-ligand complexes. The root-mean-square deviation (rmsd) of the protein backbone α-carbons is displayed in red. The rmsd of the ligand atoms is displayed in blue.

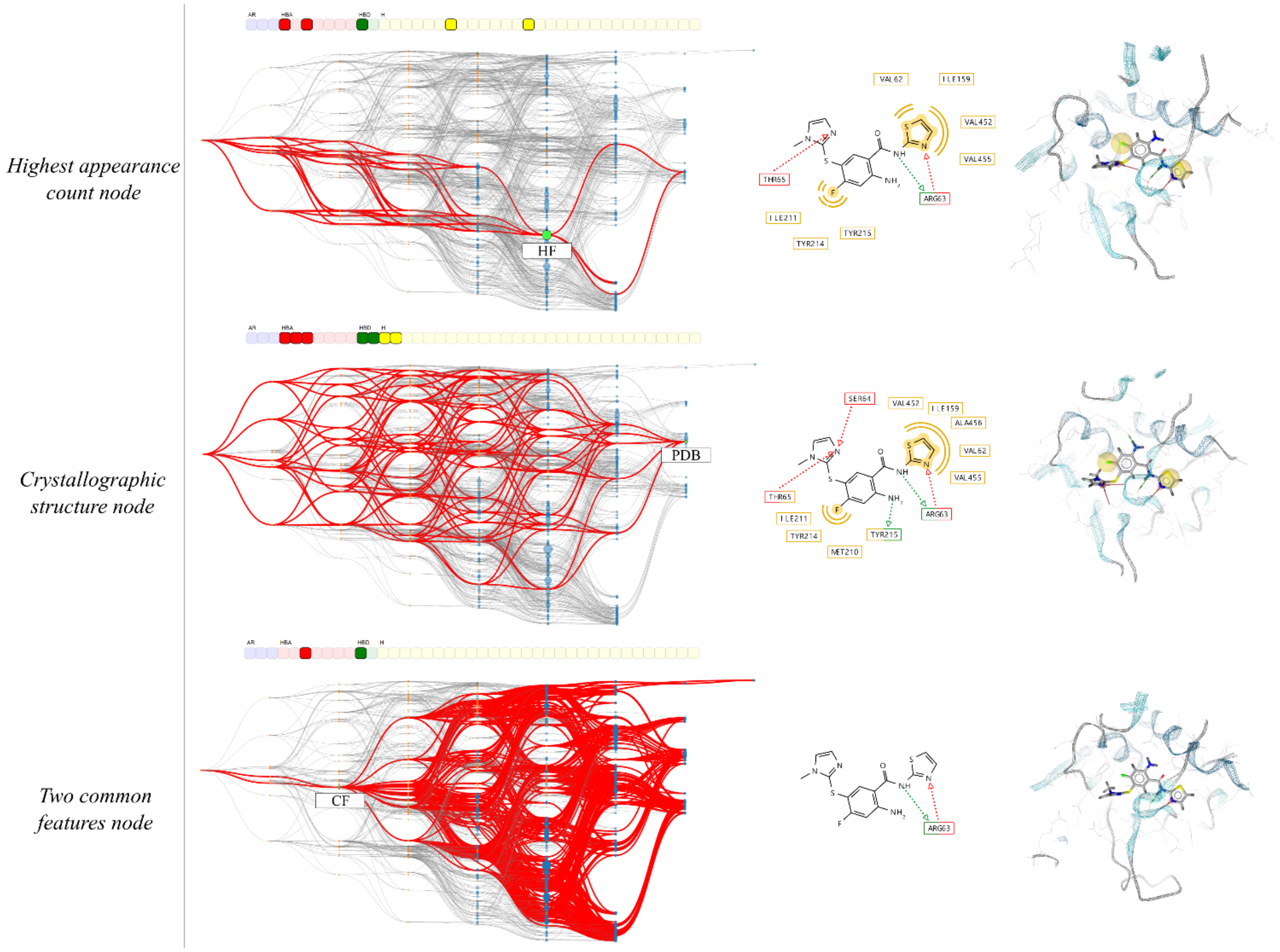

Supplementary Figure 3: Hierarchical graph of all pharmacophore models obtained from the MD simulation runs of the 1v4s system. Specific nodes were selected to highlight affiliate models. The 2D

and 3D representations of the pharmacophore models from the selected node are represented on the right part of the figure.

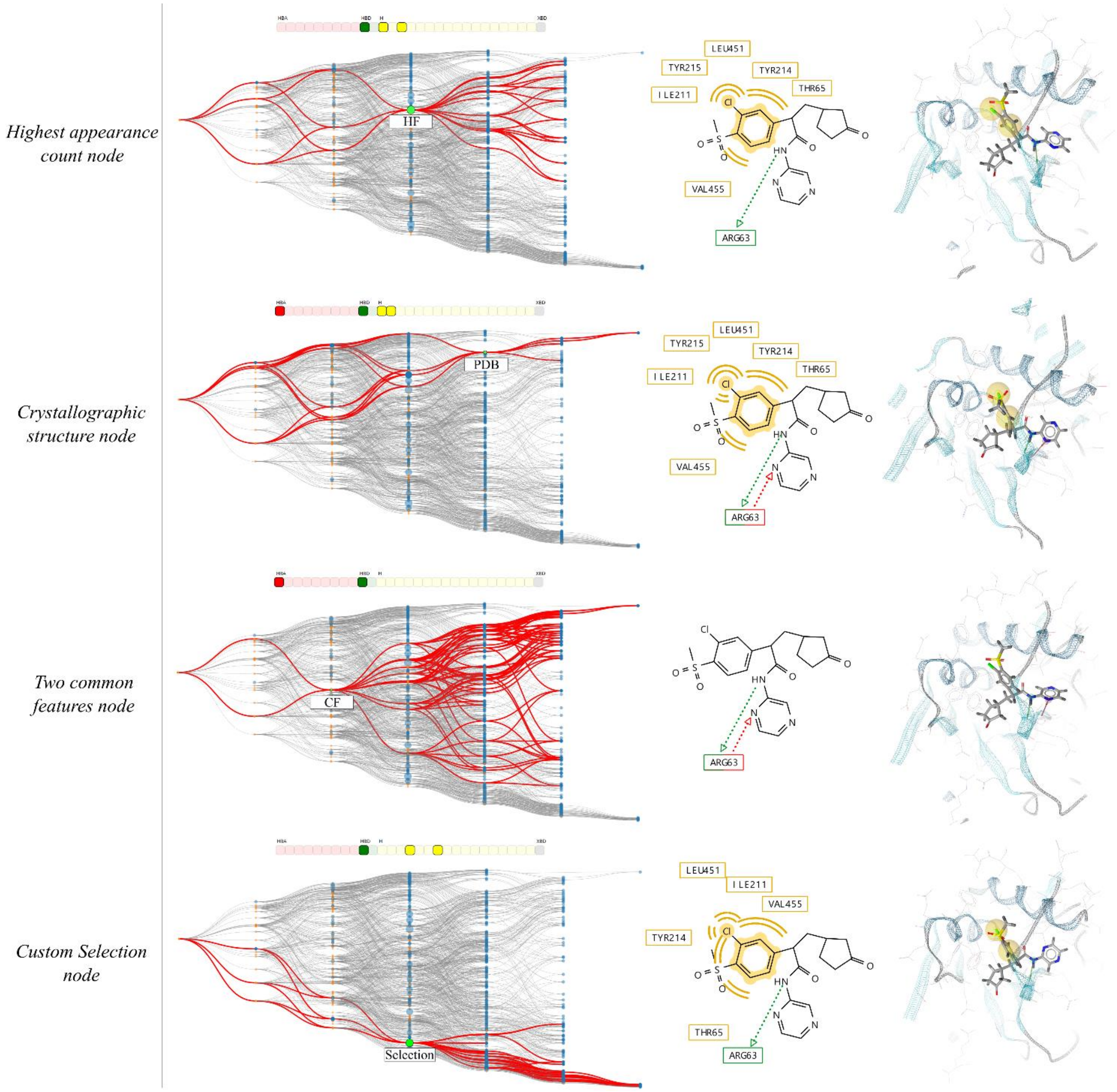

Supplementary Figure 4: Hierarchical graph of all pharmacophore models obtained from the MD simulation runs of the 4no7 system. Specific nodes were selected to highlight affiliate models. The 2D

and 3D representations of the pharmacophore models from the selected node are represented on the right part of the figure.

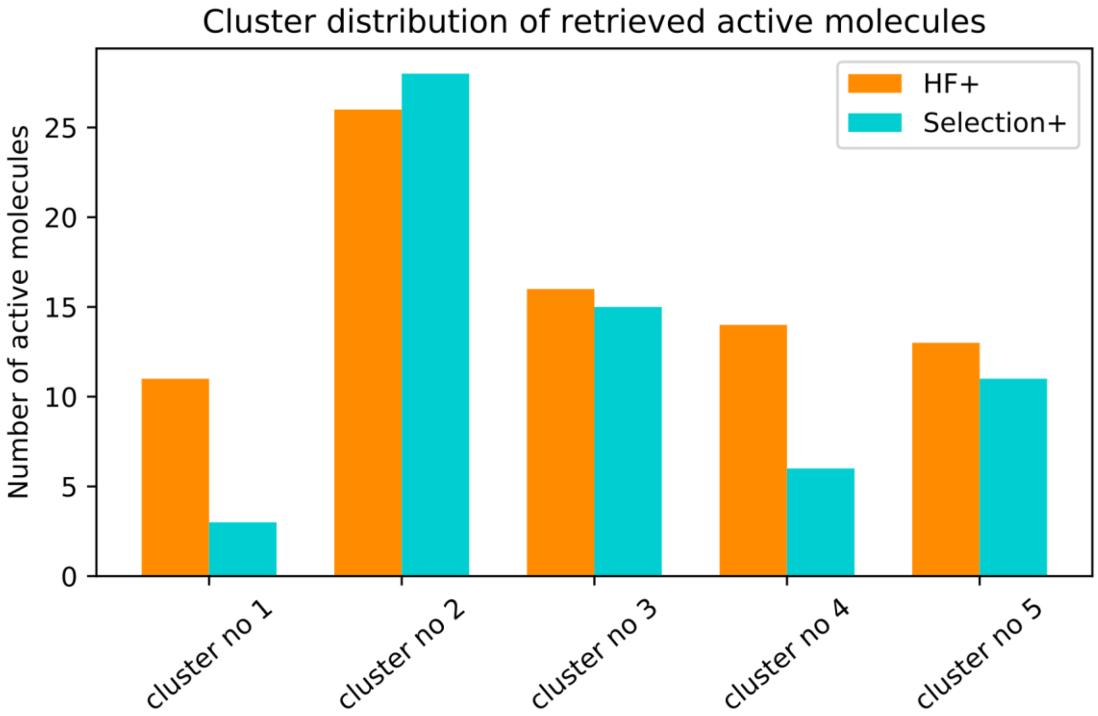

Supplementary Figure 5: Cluster distribution of retrieved active molecules at 10% of the database for the HF+ and Selection+ sets of pharmacophore models.

Crystallographic structure of 1v4s node

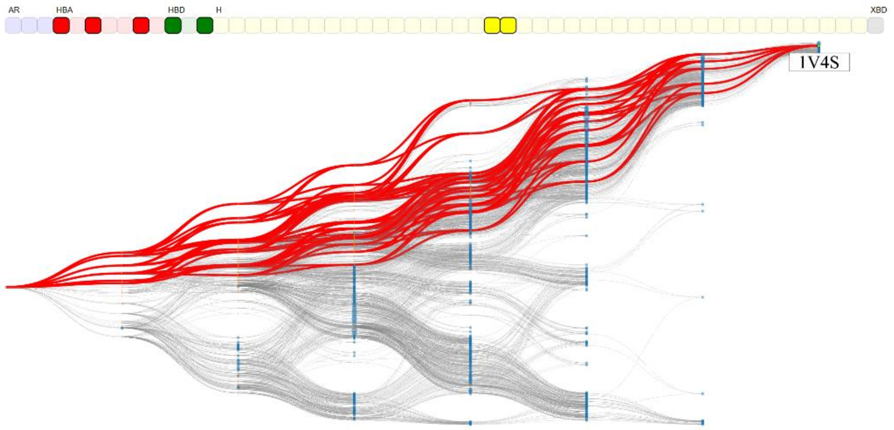

Crystallographic structure of 4no7 node

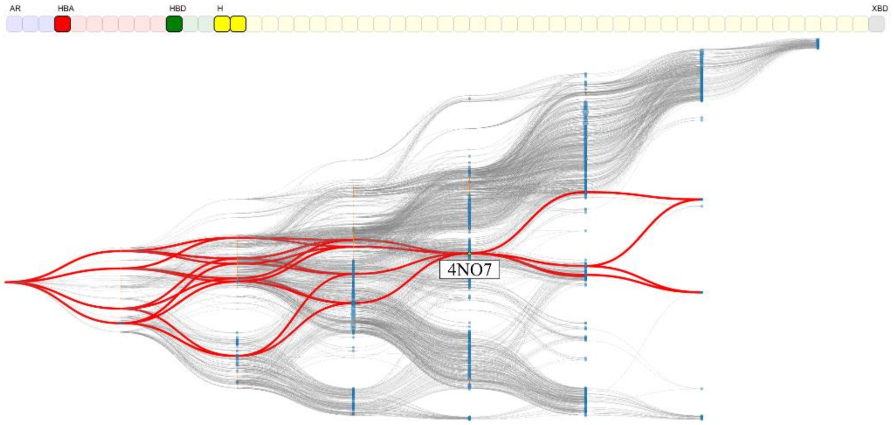

Two common features node

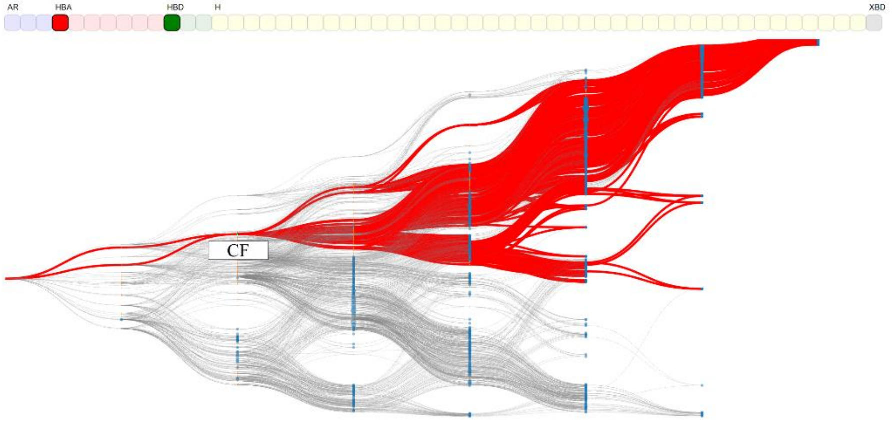

Custom Selection node

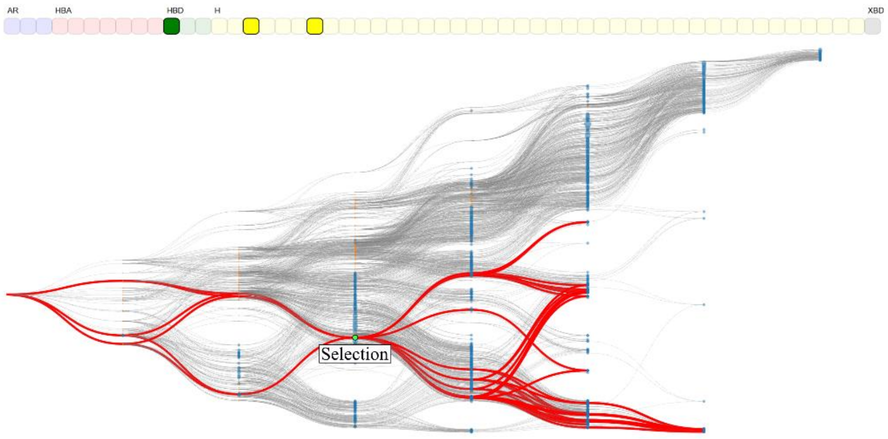

Supplementary Figure 6: Hierarchical graph of all pharmacophore models obtained from the MD simulation runs of both the 1v4s and 4no7 system. Specific nodes were selected to highlight affiliate models.

1.1 Supplementary Tables

Supplementary Table 1: Molecular dynamics simulations system information for the two protein-ligand complexes.

System	*Box size (Angstom^3)*	*Number of water molecules*	*Number of Cl- ions*	*Number of K+ ions*	*Total number of atoms*
1v4s	91.9	22818	64	86	75619
4no7	98.1	27994	79	96	91194

Supplementary Table 2: Example of active molecules for each of the 5 groups generated from ECFP similarity clustering.

Cluster number	*Number of active molecules*	*CHEMBL ID of the example molecule*	*2D structure of the example molecule*
1	97	CHEMBL556240	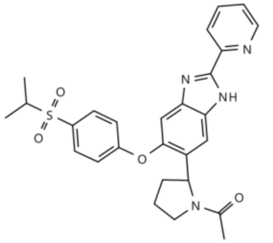
2	203	CHEMBL1165817	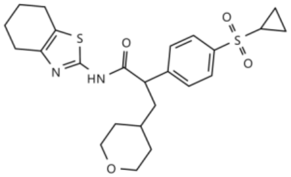
3	87	CHEMBL3221492	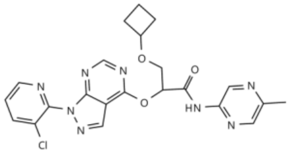
4	38	CHEMBL491322	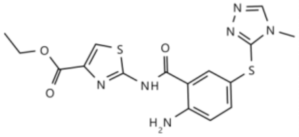
5	176	CHEMBL2408130	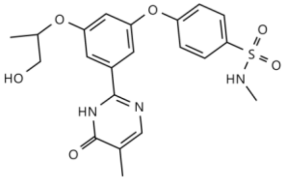

Supplementary Table 3: Details on the hierarchical graphs of pharmacophore models obtained from all the MD simulation runs, for both systems.

System	*Runs*	*Number of pharmacophore models*	*Minimum appearance count of the pharmacophore model*	*Number of unique features*	*Number of "Observed" Nodes*	*Number of "Artificial" Nodes*	*Total number of nodes*	*Variance of the projection (%)*	*Time to generate (s)*
1v4s & 4no7	1,2,3	60002	2	105	2653	2227	4880	17.0	2181
			10	55	794	623	1417	20.8	143
			30	45	349	311	660	24.5	117

Supplementary Table 4: Virtual screening results for both combined systems using different selections of pharmacophore models. The selection labeled with a + represents a subset of models used for a consensus scoring and contains every pharmacophore with at least the same features as the initial node.

System	*Selection*	*Number of common features*	*Number of pharmacophore model(s)*	*Number of hits*	*Auc at 1%*	*Auc at 5%*	*Auc at 10%*	*Auc at 50%*	*Auc at 100%*
1v4s & 4no7	CHA	0	794	12759	0.96	0.78	0.70	0.63	0.58
	CF+	2	298	11745	0.96	0.71	0.64	0.59	0.54
